# Proteolytic markers associated with a gain and loss of leg muscle mass with resistance training followed by high‐intensity interval training

**DOI:** 10.1113/EP091286

**Published:** 2023-08-17

**Authors:** J. Max Michel, Joshua S. Godwin, Daniel L. Plotkin, Paulo H. C. Mesquita, Mason C. McIntosh, Bradley A. Ruple, Cleiton A. Libardi, C. Brooks Mobley, Andreas N. Kavazis, Michael D. Roberts

**Affiliations:** ^1^ School of Kinesiology Auburn University Auburn AL USA; ^2^ Department of Physical Education Federal University of Sao Carlos Sao Carlos Brazil; ^3^ Edward Via College of Osteopathic Medicine Auburn AL USA

**Keywords:** autophagy, calpains, proteolysis, skeletal muscle, ubiquitin

## Abstract

We recently reported that vastus lateralis (VL) cross‐sectional area (CSA) increases after 7 weeks of resistance training (RT, 2 days/week), with declines occurring following 7 weeks of subsequent treadmill high‐intensity interval training (HIIT) (3 days/week). Herein, we examined the effects of this training paradigm on skeletal muscle proteolytic markers. VL biopsies were obtained from 11 untrained college‐aged males at baseline (PRE), after 7 weeks of RT (MID), and after 7 weeks of HIIT (POST). Tissues were analysed for proteolysis markers, and in vitro experiments were performed to provide additional insights. Atrogene mRNAs (*TRIM63*, *FBXO32*, *FOXO3A*) were upregulated at POST versus both PRE and MID (*P* < 0.05). 20S proteasome core protein abundance increased at POST versus PRE (*P* = 0.031) and MID (*P* = 0.049). 20S proteasome activity, and protein levels for calpain‐2 and Beclin‐1 increased at MID and POST versus PRE (*P* < 0.05). Ubiquitinated proteins showed model significance (*P* = 0.019) with non‐significant increases at MID and POST (*P* > 0.05). in vitro experiments recapitulated the training phenotype when stimulated with a hypertrophic stimulus (insulin‐like growth factor 1; IGF1) followed by a subsequent AMP‐activated protein kinase activator (5‐aminoimidazole‐4‐carboxamide ribonucleotide; AICAR), as demonstrated by larger myotube diameter in IGF1‐treated cells versus IGF1 followed by AICAR treatments (I+A; *P* = 0.017). Muscle protein synthesis (MPS) levels were also greater in IGF1‐treated versus I+A myotubes (*P* < 0.001). In summary, the loss in RT‐induced VL CSA with HIIT coincided with increases in several proteolytic markers, and sustained proteolysis may have driven this response. Moreover, while not measured in humans, we interpret our in vitro data to suggest that (unlike RT) HIIT does not stimulate MPS.

## Abstract (highlights)

- **What is the central question of this study?**
  Determining if HIIT‐induced reductions in muscle hypertrophy following a period of resistance training coincided with increases in proteolytic markers.
- **What is the main finding and its importance?**
  Several proteolytic markers were elevated during the HIIT training period implying that increases in muscle proteolysis may have played a role in HIIT‐induced reductions in muscle hypertrophy.

## 1 INTRODUCTION

It is generally appreciated that different exercise training modalities elicit unique skeletal muscle adaptations and that skeletal muscle hypertrophy occurs in response to progressive overload (i.e., resistance training; RT) (Schoenfeld, 2010; Vann et al., 2022). Owing to the plastic nature of skeletal muscle, this adaptation is not permanent given that the cessation of RT can cause an equivalent loss in skeletal muscle tissue (Seaborne et al., 2018). Endurance training can also elicit skeletal muscle adaptations such as increased mitochondrial and capillary density (Hoier & Hellsten, 2014; Mesquita et al., 2020). Such molecular adaptations have been proposed to contribute to increases in ${\dot{V}}_{O_{2}\mathrm{peak}}$, enhanced lactate clearance and higher oxidative potential (Phillips et al., 1996). High‐intensity interval training (HIIT) has become increasingly prevalent in both recreational and clinical contexts (Ellingsen et al., 2017; Reljic et al., 2019), perhaps owing to the potential for similar adaptations to steady state endurance training with a lower time cost (Burgomaster et al., 2008). HIIT is typically characterized by brief periods of intense cardiovascular exercise interspersed with rest periods (Callahan et al., 2021). Many studies have attempted to characterize muscle morphological adaptations to HIIT, with some lines of evidence suggesting hypertrophy can occur (Gahreman et al., 2016; Heydari et al., 2012; Scoubeau et al., 2022) while others suggest muscle mass is not appreciably affected (Joanisse et al., 2013, 2015).

There are both conserved and differential muscle molecular responses to RT and HIIT (Brook et al., 2022). RT has been reported to upregulate anabolic signaling markers (Pugh et al., 2015; Schiaffino et al., 2021; Schoenfeld, 2010), which provides mechanistic backing for the induction of skeletal muscle hypertrophy. Conversely, some studies indicate that HIIT can induce an anabolic response (Callahan et al., 2021), while other findings indicate that molecular responses to HIIT may interfere with anabolism or induce catabolism. For example, Haun et al. (2017) reported an increase in Muscle atrophy F‐box (MAFbx)/Atrogin‐1 and poly‐ubiquitinated protein abundance, both being markers of proteolysis via the ubiquitin–proteasome system (UPS), after three consecutive days of HIIT. Additionally, others have reported an increase in levels of muscle phosphorylated AMP‐activated protein kinase (AMPK) (Bartlett et al., 2012; de Souza et al., 2013; Torma et al., 2019), a known suppressor of anabolic signaling and upstream regulator of both muscle‐specific E3 ubiquitin ligases (e.g., muscle really interesting new gene (RING) finger 1 (MuRF1) and MAFbx) (Krawiec et al., 2007; Nakashima & Yakabe, 2007) and the autophagy/lysosomal system (Krawiec et al., 2007; Mihaylova & Shaw, 2011; Nakashima & Yakabe, 2007). In rodents there have been reports of increased *TRIM63* mRNA (coding for MuRF1), 26S proteasome activity and calpain protein abundance following 8 weeks of HIIT (Cunha et al., 2012) along with increased markers of the autophagy/lysosomal system markers after 8 and 10 weeks, respectively (Cui et al., 2019; Li et al., 2018). Nonetheless, counterevidence in rodents and humans suggests that HIIT reduces skeletal muscle atrogene expression (Castano et al., 2020; Popov et al., 2018). Taken together, it is plausible that HIIT is not as potent a stimulus as RT when considering gains in skeletal muscle mass. Furthermore, periods of RT alternating with periods of HIIT may not be able to sustain continuous gains in muscle mass.

Our laboratory recently reported that increases in vastus lateralis hypertrophy following 7 weeks of RT were lost after a subsequent 7 weeks of HIIT (Mesquita et al., 2023). Notwithstanding, the mechanisms associated with HIIT‐induced atrophy were not explored. Thus, the purpose of this study was to elucidate potential mechanisms associated with this phenotypic response. Notably, muscle biopsies were obtained from participants prior to training, following 7 weeks of RT and following 7 weeks of HIIT. Tissue was processed and assayed for markers of the UPS, the autophagy/lysosomal system and the calpain system. Additionally, in vitro analyses were performed using exercise mimetics to further explore the mechanisms promoting the observed phenotypic alterations. We hypothesized that all assayed proteolysis markers would be upregulated following 7 weeks of HIIT as compared to baseline and 7 weeks of RT.

## 2 METHODS

### 2.1 Participants and ethical approval

This study was conducted with prior review and approval from the Auburn Institutional Review Board and in accordance with the most recent revisions of the *Declaration of Helsinki* (IRB approval no.: 21‐390 MR 2109) except for being pre‐registered as a clinical trial. Notably, this is a secondary analysis of subjects from a recent study published from our laboratory (Mesquita et al., 2023).

The participants recruited for this study were young adult men from the local area that met the following criteria: (i) aged 18–30; (ii) a body mass index (BMI; body mass in kilograms/height in metres^2^) not exceeding 30 kg/m^2^; (iii) no known cardio‐metabolic disease (e.g., obesity, diabetes, hypertension, heart disease) or any condition contraindicating participation in exercise training or donating muscle biopsies; (iv) no participation in a resistance training programme more than 1 day/week for at least 2 months over the past 3 years. Following verbal and written consent, participants then completed testing procedures described in greater detail in the following sections.

### 2.2 Study design overview

Participants completed an informed consent visit, a familiarization visit, and three testing session visits to the laboratory (denoted as PRE, MID and POST). The PRE visit was conducted for baseline assessments. The MID visit occurred following 7 weeks of resistance training (2 days/week) and was ∼72 h following the last resistance exercise bout. Approximately 48–72 h following the MID visit participants took part in a 7‐week block of treadmill HIIT, and the POST visit occurred ∼72 h following the last HIIT bout. More details regarding the RT and HIIT blocks are provided in Mesquita et al. (2023). During PRE/MID/POST visits, participants were instructed to report to the laboratory following an overnight fast (≥4 h for all subjects). Furthermore, participants were instructed not to modify their dietary patterns throughout the study, and no dietary supplements were administered throughout the duration of the intervention.

### 2.3 PRE/MID/POST testing sessions

#### 2.3.1 Hydration testing

During the PRE/MID/POST testing sessions participants donated a urine sample (∼5 ml) that was assessed for urine specific gravity (USG) testing using a handheld refractometer (Atago; Bellevue, WA, USA). All participants had a USG level ≤1.020, which was used as a threshold of sufficient hydration to continue testing (American College of Sports Medicine et al., 2007).

#### 2.3.2 Body composition and vastus lateralis assessments using ultrasound

Height and body mass were evaluated using a digital column scale (Seca 769; Hanover, MD, USA). Height was measured to the closest 0.5 cm and body mass was measured to the closest 0.1 kg. Participants were then subjected to a full body dual energy X‐ray absorptiometry (DXA) scan (Lunar Prodigy; GE Healthcare, Chicago, IL, USA), and following DXA scans vastus lateralis muscle cross‐sectional area was assessed using an ultrasound device (NextGen LOGIQe R8, GE Healthcare) with a multifrequency linear‐array transducer as previously described by our lab (Ruple et al., 2022) (L4‐12T, 4–12 MHz, GE Healthcare). Briefly, a semi‐rigid pad placed around the thigh and secured with an adjustable strap was used as a guide to ensure movement of the probe occurred in a uniform transverse plane. Imaging began on the lateral aspect of the thigh and moved medially until the rectus femoris was visualized. These images were captured using the panoramic function of the device. VL CSA was calculated by manual tracing of the border of the VL along the fascia using the polygon function in freely available ImageJ software (National Institutes of Health, Bethesda, MD, USA). Images were taken by the same blinded investigator at all three time points. Previously determined test–retest reliability for VL CSA yielded an intra‐class correlation coefficient of 0.99, standard error of the measurement of 1.57 cm^2^, and minimal difference of 3.08 cm^2^ (Ruple et al., 2023). Further details of these testing procedures can be found in the parent publication, Mesquita et al. (2023).

#### 2.3.3 Vastus lateralis biopsies

After ultrasound images were obtained, skeletal muscle biopsies were collected from the right vastus lateralis at the marked location from ultrasound imaging as described by our laboratory prior (Ruple et al., 2021; Sexton et al., 2021). Muscle used for the RNA and protein work herein (∼30–50 mg) was placed in pre‐labeled foils and flash‐frozen in liquid nitrogen within 2 min of the biopsy. Muscle used for immunohistochemistry (IHC; ∼20–30 mg) was preserved in freezing medium for histology (Tissue‐Tek; Sakura Finetek Inc., Torrence, CA, USA), slow‐frozen in liquid nitrogen‐cooled isopentane and subsequently stored at −80°C. Notably, MID and POST biopsies were obtained ∼2 cm proximal of each preceding biopsy scar. Notably, muscle biopsies were collected from 11 participants at all three time points, and thus the following analyses are limited to *n* = 11 subjects.

### 2.4 Wet laboratory analyses

#### 2.4.1 Real‐time qPCR

Frozen muscle foils were removed from −80°C storage and crushed with a liquid nitrogen‐cooled ceramic mortar and pestle. Approximately 10 mg of muscle was used to isolate RNA via the Ribozol method per the manufacturer's recommendations (VWR, Randor, PA, USA). Following RNA isolation, the RNA pellet was reconstituted in 20 μl of RNase‐free water and RNA concentrations were determined in duplicate at an absorbance of 260 nm by using a NanoDrop Lite (Thermo Scientific, Waltham, MA, USA). Thereafter, cDNA (2 μg) was synthesized using a commercial qScript cDNA SuperMix (Quanta Biosciences, Gaithersburg, MD, USA) per the manufacturer's recommendations.

qPCR was performed with gene‐specific primers and SYBR‐green‐based methods (Quanta Biosciences) with gene‐specific primers designed with primer design software (Primer3Plus, Cambridge, MA, USA) using a real‐time PCR thermal cycler (Bio‐Rad Laboratories, Hercules, CA, USA). The final volume of qPCR reactions was 20 μl, which contained a final concentration of 2 μM of forward and reverse primers and 25 ng of cDNA. All reactions were performed in duplicate. Forward and reverse sequences for all genes are shown in Table 1. Fold‐change values from PRE were performed using the 2^ΔΔ^ *^C^* ^q^ method where 2^Δ^ *^C^* ^q^ = 2^[housekeeping gene geometric mean (HKG)^ *^C^* ^q − gene of interest^ *^C^* ^q]^, and 2^ΔΔ^ *^C^* ^q^ (or fold‐change) = [2^Δ^ *^C^* ^q^ individual PRE, MID or POST value/2^Δ^ *^C^* ^q^ average of all PRE values]. Primer sets for glyceraldehyde‐3‐phosphate dehydrogenase and valosin‐containing protein were used for mRNA normalization. Notably, the geometric means of these two targets were stable across all time points.

**TABLE 1:** Human primer sequences used for qPCR.

Primer	Forward primer sequence (5′ → 3′)	Reverse primer sequence (5′ → 3′)
*TRIM63*	CTCAGTGTCCATGTCTGGAGGCCGTT	GGCCGACTGGAGCACTCCTGTTTGTA
*FBXO32*	TATTGCACCCTGGGGGAAGCTTTCAA	TCCAACAGCCGGACCACGTAGTTAAA
*FOXO3A*	GGGGAACTTCACTGGTGCTA	TGTCCACTTGCTGAGAGCAG
*GAPDH* ^a^	AACCTGCCAAATATFATGAC	TCATACCAGGAAATGAGCTT
*VCP* ^a^	TGGCATGACTCCCTCCAAAG	CAGCTCAFFACCCTTGATCG

^a^ The geometric mean of *GAPDH* and *VCP* was used to normalize mRNA expression for *TRIM63*, *FBXO32* and *FOXO3A*. Abbreviations: FBXO32, F‐box protein 32; FOXO3A, Forkhead box O3a; GAPDH, glyceraldehyde‐3‐phosphate dehydrogenase; TRIM63, tripartite motif containing 63; VCP, valosin‐containing protein.

#### Western blotting

During tissue extraction for RNA isolation, a portion of muscle tissue was also allocated for protein isolation. Approximately 20 mg of tissue was placed in 1.7 ml microcentrifuge tubes and homogenized in a sucrose homogenization buffer using a glass Dounce homogenizer according to Spinazzi et al. (2012). Tissues were then centrifuged at 600 *g* for 10 min.

Lysates were batch process‐assayed for total protein content using a BCA Protein Assay Kit (Thermo Fisher Scientific). Lysates (*n* = 11) were then prepared for western blotting using 4× Laemmli buffer at 1 μg/μl. Thereafter, 15 μl of prepped samples were loaded onto 4−15% SDS‐polyacrylamide gels (Bio‐Rad) and subjected to electrophoresis (180 V for 50 min) using pre‐made 1× SDS‐PAGE running buffer (VWR). Proteins were then transferred (200 mA for 2 h) to polyvinylidene difluoride membranes (Bio‐Rad), Ponceau stained and imaged to ensure equal protein loading between lanes. Membranes were then blocked for 1 h at room temperature with 5% non‐fat milk powder in Tris‐buffered saline with 0.1% Tween‐20 (TBST; VWR). Membranes containing 1‐h treated samples were incubated with the following antibodies at a 1:1000 dilution in TBST with 5% bovine serum albumin (BSA) overnight: rabbit anti‐20S core subunit antibody cocktail (cat. no.: BML‐PW8155; Enzo Life Sciences; Farmingdale, NY, USA), rabbit anti‐calpain‐1 (cat. no.: 2556; Cell Signaling Technology, Danvers, MA, USA), rabbit anti‐calpain‐2 (cat. no.: 70655; Cell Signaling), rabbit anti‐ubiquitin (cat. no.: 3933; Cell Signaling), rabbit anti‐LC3B (cat. no.: 2775; Cell Signaling), rabbit anti‐phosphorylated AMPKα (Thr172) (cat. no.: 2535; Cell Signaling), rabbit anti‐pan AMPKα (cat. no.: 5831; Cell Signaling), rabbit anti‐phosphorylated Unc‐51‐like kinase 1 (ULK1) (Ser555) (cat. no.: 5869; Cell Signaling), rabbit anti‐pan ULK1 (cat. no.: 8054; Cell Signaling), rabbit anti‐Beclin‐1 (cat. no.: NB110‐8731855; Novus Biologicals, Centennial, CO, USA), and mouse anti‐puromycin (1:10,000 dilution; cell culture lysate only) (cat. no.: MABE342; Millipore Sigma, Burlington, MA, USA). The following day, membranes were incubated with horseradish peroxidase‐conjugated anti‐rabbit antibodies (1:2000; Cell Signaling) in TBST with 5% BSA at room temperature for 1 h. Membrane development was performed using an enhanced chemiluminescence reagent (Luminata Forte HRP substrate; Millipore Sigma), and band densitometry was performed using a gel documentation system and associated densitometry software (ChemiDoc Touch, Bio‐Rad). Densitometry values of protein targets were normalized to Ponceau densities. These values were then normalized to PRE values that were averaged to a value of 1.00 and data were expressed as relative expression units (REUs). Phosphorylated target band densities were divided by the pan densities of these targets to obtain a ratio.

#### 2.4.2 Calpain and 20S proteasome activity assays

Calpain and proteasome activity assays were performed in accordance with previously published methods from our laboratory (Osburn et al., 2021). Briefly, 20S proteasome activity assays were performed using commercially available fluorometric kits (cat. no.: APT280; Millipore Sigma) per the manufacturer's instructions. Lysates (40 μl) were loaded in duplicate onto black 96‐well plates with the enzyme mix provided by the kit and incubated at 37°C for 60 min. Fluorescence was then read using a microplate fluorometer (BioTek Synergy H1, Winooski, VT, USA) using 380 nm excitation and 460 nm emission settings. Fluorometric readings were divided by total protein loaded per well (i.e., RFU per μg muscle soluble protein). Calpain activity assays were performed using commercially available fluorometric kits (cat. no.: ab65308; Abcam, Cambridge, UK). Lysates (10 μl) were loaded in duplicate onto black 96‐well plates with the enzyme mix provided by the kit and incubated at 37°C for 60 min. Fluorescence was then read using a microplate fluorometer (BioTek Synergy H1) using 400 nm excitation and 505 nm emission settings. Again, fluorometric readings were divided by total protein loaded per well and expressed as relative fluoresence units (RFU) per μg muscle soluble protein. The average coefficient of variation values for duplicate proteasome activity and calpain activity data were 1.94% and 2.41%, respectively.

#### 2.4.3 IHC for phalloidin‐actin staining

Sections from OCT‐preserved samples were sectioned at a thickness of 7 μm using a cryotome (Leica Biosystems, Buffalo Grove, IL, USA) and adhered to positively charged histology slides. Slides were then stored at −80°C until batch processing occurred for phalloidin–actin staining. Due to sample constraints, OCT‐preserved tissue at all three time points were only available for 10 participants and therefore this analysis is restricted to *n* = 10 subjects.

For the determination of myofibril area and relative actin content per myofibre, F‐actin labelling using Alexa Flour 594 (AF594)‐conjugated phalloidin was performed to determine myofibril area and relative actin content per myofibre. Briefly, serial sections were allowed to air dry for ∼1.5–2 h at room temperature followed by fixation in chilled acetone (−20°C) for 5 min. Sections were then incubated with 3% H_2_O_2_ for 15 min and true black (cat. no.: 230007; Biotium, Fremont, CA, USA) for 1 min to quench auto‐fluorescence. Sections were then blocked with 2.5% normal horse serum/5% normal goat serum for 1 h. Sections were incubated overnight at 4°C with mouse anti‐dystrophin MANDY S8 (1:20) (cat. no.: 8H11; DSHB, Iowa City, IA, USA). The following morning, sections were incubated for 1 h with phalloidin conjugated to AF594 (1:100) (cat. no.: A12381; Thermo Fisher Scientific) and Alexa Fluor 488‐conjugated anti‐mouse IgG1 (1:250) (cat. no.: A11001; Thermo Fisher Scientific). Sections were then incubated in 4′,6‐diamidino‐2‐phenylindole (DAPI; 1:10,000; cat. no.: D3571, Thermo Fisher Scientific) for 15 min. Slides were then mounted with glass coverslips using 50/50 phosphate‐buffered saline (PBS) + glycerol. Digital images were captured using a fluorescence microscope (Nikon Instruments, Melville, NY, USA) using a ×20 objective. According to previous reports, the quantification of myofibril area per fibre was performed using ImageJ software (Fox et al., 2021; Ruple et al., 2021). Briefly, the relative scale within ImageJ was set such that 1 pixel was equivalent to 0.451 μm, images were then split into RGB channels, and the red, phalloidin‐containing channel was converted to greyscale. The threshold function was then used to generate a binary black and white image of stained versus unstained portions of fibres. Fibres were then traced, and myofibril areas were provided as a percentage per fibre area. These values were reported as the percentage of myofibre occupied by the myofibril or as total contractile protein per fibre (i.e., % myofibril per myofibre × fibre cross‐sectional area).

### 2.5 In vitro experimentation

Immortalized C2C12 murine muscle cells were purchased from the American Type Culture Collection (ATCC, Manassas, VA, USA), and all incubations occurred at 37°C in an atmosphere containing 5% CO_2_–95% room air. Myoblasts (passage 5) were seeded on six‐well plates at a density of 3.0 × 10^5^ in 3 ml/well of growth medium (GM), consisting of Dulbecco's modified Eagle's medium (DMEM; Corning, Corning, NY, USA) supplemented with 10% fetal bovine serum (Corning) and 1% penicillin/streptomycin (VWR). When cells reached ∼80–90% confluence, differentiation was induced by switching to differentiation medium (DM), which consisted of DMEM supplemented with 2% horse serum (VWR). DM was replaced daily for 3 days, then every 36–48 h until the end of differentiation (day 7). Thereafter, treatments occurred as described below. On the day of treatments, cells were rinsed with Dulbecco's phosphate buffered saline (without calcium and magnesium; Corning), then serum‐starved (treated with DMEM only) for 1 h. Cells were then treated for 24 h with DM containing the following (*n* = 9 replicates per treatment): (i) dimethyl sulfoxide (DMSO) vehicle (0.1% DMSO) (DMSO; cat. no. 25‐950‐CQC; Corning); (ii) 200 ng/ml insulin‐like growth factor 1 (IGF1; cat. no. 791‐MG; R&D Systems; Minneapolis, MN, USA); (iii) 1 mM 5‐aminoimidazole‐4‐carboxamide‐1‐β‐d‐ribofuranoside (AICAR; cat. no.: A2528; Tokyo Chemical Industry Corp., Tokyo, Japan); and (iv) 200 ng/ml IGF1 followed by a 24‐h treatment with 1 mM AICAR (24 h IGF1 followed by 24 h AICAR for a 48 h total treatment; I+A). A dose of 200 ng/ml IGF1 was chosen based on Valentino et al. (2021) who used a similar dose to stimulate hypertrophy of C2C12 myotubes. A dose of 1 mM AICAR was chosen based on its common usage in stimulating AMPK activity (Hinkle et al., 2022; Sato et al., 2014; Tong et al., 2009). DMSO vehicle was chosen due to the necessary reconstitution of AICAR in DMSO to create a stock solution. It was determined that 0.1% DMSO was the final concentration within the AICAR treatments, and thus the 0.1% DMSO vehicle was used as a control to parse out any confounding effects of DMSO on C2C12 cells.

Approximately 15 min prior to collection, cells were pulse‐labelled with 1 mM puromycin dihydrochloride (cat. no.: 97064–280; VWR) in PBS for subsequent muscle protein synthesis (MPS) assessment using the surface sensing of translation (SUnSET) method as performed previously in our laboratory (Mobley et al., 2017). Upon collection, cells were washed with PBS and lysed using 300 ml of ice‐cold cell lysis buffer (20 mM Tris–HCL (pH 7.5), 150 mM NaCl, 1 mM Na_2_EDTA, 1 mM EGTA, 1% Triton, 2.5 mM sodium pyrophosphate, 1 mM β‐glycerophosphate, 1 mM Na_3_VO_4_, 1 mg/ml leupeptin (Cell Signaling)) pre‐stocked with protease and Tyr/Ser/Thr phosphatase inhibitors. Cell lysates were placed in 1.7 ml microtubes and stored at −80°C until protein concentration determination.

Lysates were batch‐process‐assayed for total protein content using a commercially available BCA protein assay kit (Thermo Fisher Scientific). Lysates were then prepared for western blotting as described above, and values were normalized to DMSO control values where the average for this treatment was set to 1.00, and data are expressed as relative expression units (REUs). Similarly, calpain and 20S proteasome activity assays were performed as described above.

### 2.6 Cytology staining

Cells from each treatment group (*n* = 3) were stained for morphological assessment. Briefly, cells were fixed with 4% paraformaldehyde for 15 min, then rinsed in PBS containing 0.2% Triton X‐100 (PBS/Triton) for 3 × 3 min washes. Following initial permeabilization, cells were blocked in 1% BSA (in PBS/Triton) for 1 h at room temperature with gentle agitation. After blocking, cells were once again washed in PBS/Triton. Cells were then incubated for 3 h with a 1:100 dilution of a primary antibody against sarcomeric myosin (cat. no.: A4.1025, DSHB) at room temperature with gentle agitation. Following primary incubation, cells were washed in PBS/Triton and further incubated with a 1:250 dilution of a goat anti‐mouse IgG secondary antibody (cat. no.: A‐11001; Thermo Fisher Scientific) conjugated to AF488 for 1 h at room temperature with gentle agitation. Cells were washed with PBS/Triton before incubating for 10 min with DAPI (cat. no.: D3571; Thermo Fisher Scientific). After incubation with DAPI, cells were again washed in PBS/Triton before adding a coverslip mounted with PBS/glycerol. Cells were then imaged at ×10 and analysed for myotube morphology using ImageJ software similar to a previous study in our laboratory (Osburn et al., 2021). A conversion factor of 0.964 mm/pixel was applied and the myotube diameter was obtained using the straight‐line function. Myotube diameter was obtained from three points along the myotube for as many myotubes as were visible in each field of view, and this resulted in an image‐wide average for myotube diameter.

### 2.7 Statistics

Statistical analyses were performed using GraphPad Prism (Version 9.3.1; GraphPad Software, San Diego, CA, USA). All data in tables and figures are presented as means ± standard deviation (SD), and individual respondent data are also presented.

All human data were checked for normality using the Shapiro–Wilk test. For normally distributed data, one‐way repeated measures ANOVA was performed. In cases where model significance was obtained (*P* < 0.05), Tukey *post hoc* test was performed between time points. For non‐normally distributed data, Friedman's tests was performed. In cases where model significance was obtained (*P* < 0.05), Wilcoxon's signed rank test was performed between time points.

In vitro data were checked for normality using the Shapiro–Wilk test. For normally distributed data, one‐way ANOVA was performed. In cases where model significance was obtained (*P* < 0.05), Tukey's *post hoc* test was performed. For non‐normally distributed data, the Kruskal–Wallis test was performed. In cases where model significance was obtained (*P* < 0.05), the Mann–Whitney *U*‐test was performed between treatments.

## 3 RESULTS

### 3.1 Participant characteristics

Baseline participant characteristics are presented in table form by Mesquita et al. (2023). Briefly, 11 college‐aged males (23 ± 4 years) volunteered for this study. At PRE, participants weighed 80.8 ± 17.1 kg with 57.4 ± 9.0 kg being lean/soft tissue mass and 20.7 ± 9.9% being body fat percentage.

### 3.2 Muscle morphology and strength adaptations

**FIGURE 1:**
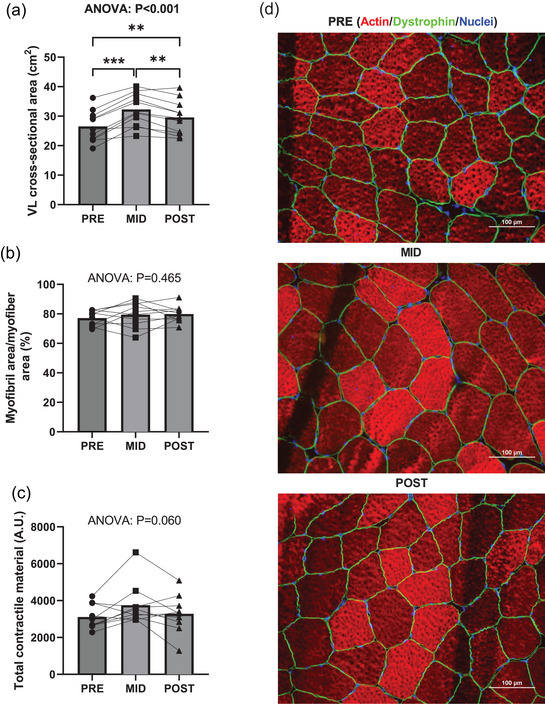
Hypertrophy–atrophy phenotype with RT followed by HIIT. (a–c) Data presented as bar graphs with individual data points and standard deviation as error bars (*n* = 10 subjects; 3 time points) for vastus lateralis cross‐sectional area (a), area of the myofibre occupied by myofibrils (b), and total contractile area per myofibre (c). (d) Representative ×20 objective images of immunohistochemistry staining (scale bar = 100 μm). Statistical significance is denoted by **P < 0.01, ***P < 0.001. A.U., arbitrary units.

Ultrasonography (US)‐derived VL cross‐sectional area (CSA) demonstrated model significance (*P* < 0.001); Figure 1a), with increases from PRE to MID (*P* < 0.001) followed by subsequent declines from MID to POST (*P* = 0.001), with POST being greater than PRE (*P* = 0.008). The percentage of myofibres occupied by myofibrillar protein did not demonstrate model significance (*P* = 0.465; Figure 1b). Similarly, total contractile protein (percentage of the myofibre occupied by myofibrils × fibre cross‐sectional area) did not demonstrate model significance (*P* = 0.060, Figure 1c). Representative images of phalloidin staining are included in Figure 1d. Mixed and fibre type specific fibre cross‐sectional area data can be found in Mesquita et al. (2023).

### 3.3 Adaptations in ubiquitin‐proteasome markers

**FIGURE 2:**
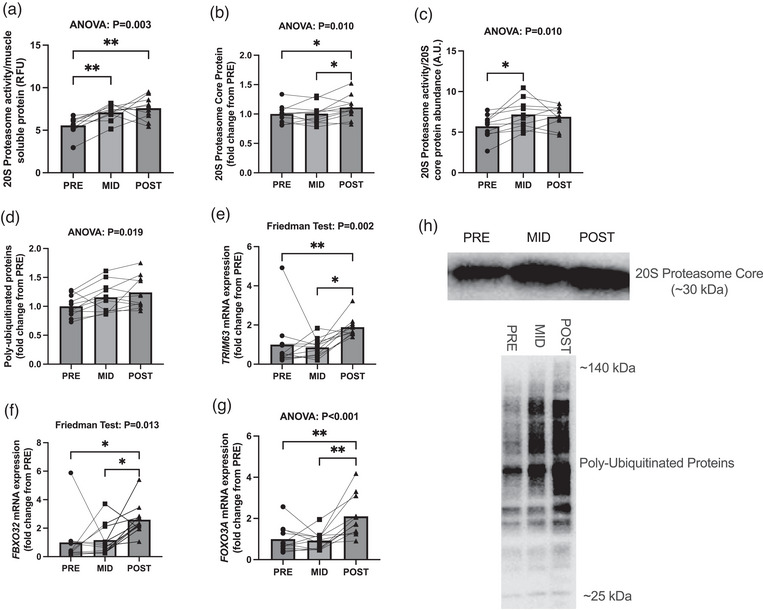
Ubiquitin–proteasome markers with RT followed by HIIT. (a–g) Data are presented as bar graphs with individual data points and standard deviation as error bars (*n* = 11 subjects, 3 time points for western blots; *n* = 10 subjects, 3 time points for activity assay) for 20S proteasome activity normalized to muscle protein (a), 20S proteasome protein expression (b), 20S proteasome activity normalized to proteasome protein levels (c), poly‐ubiquitinated protein abundance (d), *TRIM63*/Atrogin‐1 mRNA expression (e), *FBXO32*/MuRF1 mRNA expression (f), *FOXO3A* mRNA expression (g). (h) Representative western blot images for 20S proteasome and poly‐ubiquitinated proteins. Statistical significance is denoted by *P < 0.05, **P < 0.01. A.U., arbitrary units; RFU, relative fluorescence units.

There was model significance for 20S proteasome activity normalized to muscle soluble protein (*P* = 0.003; Figure 2a), where significant increases were observed from PRE to MID (*P* = 0.001) and PRE to POST (*P* = 0.004), with no differences observed from MID to POST (*P* = 0.650). There was also model significance for 20S proteasome core abundance (*P* = 0.003 Figure 2b), where POST was greater than both PRE (*P* = 0.031) and MID (*P* = 0.049), with no differences observed from PRE to MID (*P* = 0.985). Analysis of 20S proteasome activity normalized to 20S proteasome core abundance demonstrated model significance (*P* = 0.010; Figure 2c), with increases being observed from PRE to MID (*P* = 0.012), and no differences seen from PRE to POST (*P* = 0.055) or MID to POST (*P* = 0.823). There was model significance for poly‐ubiquitinated proteins (*P* = 0.019; Figure 2d), with no differences in PRE versus MID (*P* = 0.069), MID versus POST (*P* = 0.422), or PRE versus POST (0.053). *TRIM63* mRNA demonstrated model significance (*P* = 0.002; Figure 2e) where POST was greater than both PRE (*P* = 0.004) and MID (*P* = 0.032) with no differences in PRE and MID (*P* > 0.999)*. FBXO32* mRNA similarly demonstrated model significance (*P* = 0.013; Figure 2f) where POST was greater than both PRE (*P* = 0.032) and MID (*P* = 0.032) with no differences in PRE and MID (*P* > 0.999)*. FOXO3A* mRNA demonstrated model significance (*P* < 0.001; Figure 2g), where POST was greater than both PRE (*P* = 0.006) and MID (*P* = 0.004) with no difference in PRE and MID (*P* = 0.928).

### 3.4 Adaptations in calpain markers

**FIGURE 3:**
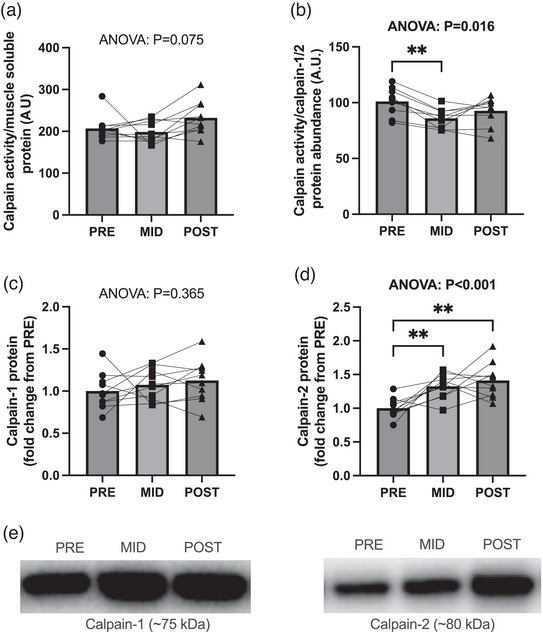
Calpain markers with RT followed by HIIT. (a–d) Data are presented as bar graphs with individual data points and standard deviation as error bars (*n* = 10 subjects; 3 time points) for calpain activity normalized to muscle protein (a), calpain activity normalized calpain‐1 and calpain‐2 protein abundance (b), calpain‐1 protein abundance (c), calpain‐2 protein abundance (d). (e) Representative images of calpain‐1 and calpain‐2 western blots. Statistical significance is denoted by *P < 0.05, **P < 0.01. A.U., arbitrary units.

Calpain activity normalized to muscle soluble protein did not demonstrate model significance (*P* = 0.075; Figure 3a). Calpain activity normalized to calpain‐1/2 protein abundance demonstrated model significance (*P* = 0.016, Figure 3b), with MID being lower than PRE (*P* = 0.001), but no differences were observed between POST and PRE (*P* = 0.282) or MID (*P* = 0.270). Calpain‐1 protein abundance did not demonstrate model significance (*P* = 0.365; Figure 3c) nor were any between‐time‐point differences revealed (*P* ≥ 0.472). Conversely, calpain‐2 protein abundance showed model significance (*P* < 0.001; Figure 3d), with PRE being less than both MID (*P* = 0.009) and POST (*P* = 0.003), while no differences were observed from MID to POST (*P* = 0.679).

### 3.5 Adaptations in autophagy/lysosomal markers

**FIGURE 4:**
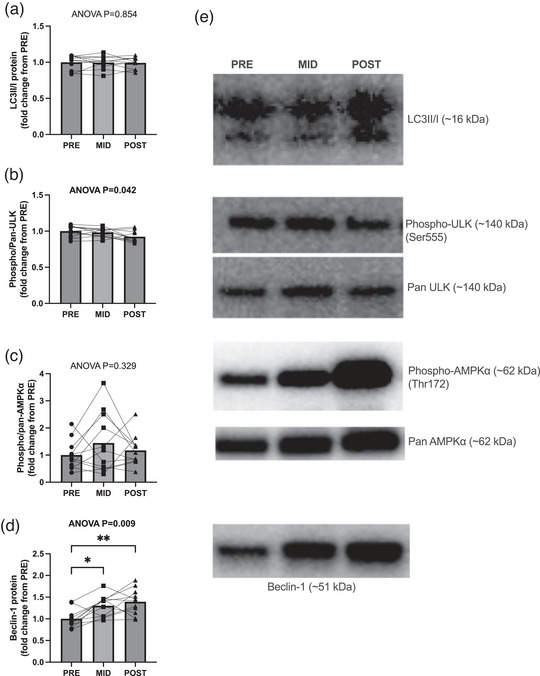
Autophagy‐lysosomal markers with RT followed by HIIT. (a–d) Data are presented as bar graphs with individual data points and standard deviation as error bars (*n* = 11 subjects; 3 time points) for LC3II/I protein expression (a), phosphorylated‐ULK/pan‐ULK protein expression (b), phosphorylated‐AMPKα/pan‐AMPKα protein expression (c), and Beclin‐1 protein expression (d). (e) Representative western blot images for LC3II/I, phosphorylated‐ULK, pan‐ULK, phosphorylated AMPKα, pan‐AMPKα and Beclin‐1. Statistical significance is denoted by *P < 0.05, **P < 0.01.

LC3II/I ratio, a marker of autophagic flux, did not reach model significance (*P* = 0.854; Figure 4a), nor were any between‐time‐point differences observed (*P* > 0.804). Phosphorylated ULK1/pan ULK1 demonstrated model significance (*P* = 0.042; Figure 4b); however, no between‐time‐point differences were observed (*P* > 0.105). Phosphorylated AMPK/pan AMPK did not exhibit model significance (*P* = 0.329; Figure 4c), nor were any between‐time‐point differences observed (*P* > 0.350). Beclin‐1 protein abundance showed model significance (*P* = 0.009; Figure 4d), with PRE being less than both MID (*P* = 0.012) and POST (*P* = 0.005), and no differences seen between MID and POST (*P* = 0.601).

### 3.6 In vitro experiments

**FIGURE 5:**
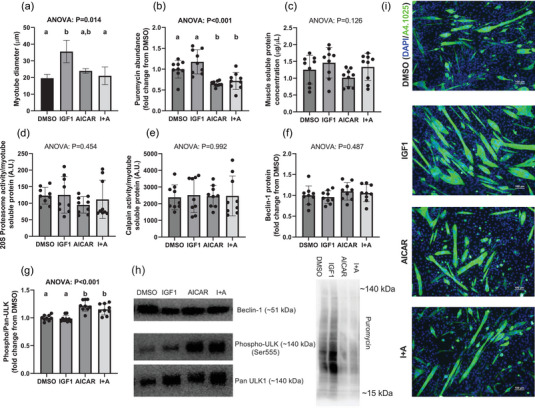
In vitro experiments. (a–g) Data are presented as bar graphs with standard deviation error bars (*n* = 3 replicates per treatment for cytology staining; *n* = 9 replicates per treatment for cell lysate collection) for myotube diameter (a), puromycin abundance (b), muscle soluble protein concentration (c), 20S proteasome activity (d), calpain activity (e), Beclin‐1 protein abundance (f), and phosphorylated/pan ULK‐1 (g). (h) Representative images western blot images for Beclin‐1, phospho‐ULK, pan ULK. (i) ×10 representative images of cytology staining (scale bar = 100 μm). Bars that do not share the same superscript letter are statistically different (*P* < 0.05). I+A, IGF1 treatments followed by AICAR treatments (24‐h treatments per compound). A4.1025, antibody against myosin heavy chain; AICAR, 5‐aminoimidazole‐4‐carboxamide ribonucleotide (24‐h treatments); DAPI, 4,6‐diamidino‐2 phenylindole; DMSO, dimethyl sulfoxide (non‐treated cells); IGF1, insulin‐like growth factor 1 (24‐h treatments).

Myotube diameter demonstrated model significance (*P* = 0.014; Figure 5a) with IGF1 having a larger myotube diameter than both DMSO (*P* = 0.026) and I+A (*P* = 0.017), but not AICAR (*P* = 0.068). Myotube diameters of DMSO (*P* = 0.984) and AICAR (*P* = 0.853) did not differ from I+A (*P* = 0.752), nor did DMSO differ from AICAR (*P* = 0.752). Puromycin incorporation, assessed via immunoblotting, demonstrated model significance (*P* < 0.001; Figure 5b), with IGF1 and being significantly greater than both AICAR (*P* < 0.001) and I+A (*P* < 0.001) with no difference from DMSO (*P* = 0.300). DMSO demonstrated significantly greater puromycin incorporation than AICAR (*P* = 0.006) and I+A (*P* = 0.034), while AICAR was not different from I+A (*P* = 0.902). Total muscle soluble protein (*P* = 0.126), 20S proteasome core activity (*P* = 0.454), calpain activity (*P* = 0.992) and Beclin‐1 protein abundance (*P* = 0.487) all did not demonstrate model significance (Figure 5c–f). Phosphorylated/pan ULK1 demonstrated model significance (*P* < 0.001; Figure 5g), with I+A being greater than both DMSO (*P* = 0.002) and IGF1 (*P* < 0.001), AICAR being greater than DMSO (*P* < 0.001) and IGF1 (*P* < 0.001), with no differences between I+A versus AICAR (*P* = 326) nor IGF1 versus DMSO (*P* = 987).

## 4 DISCUSSION

Primary findings from the current study include: (i) multiple markers of the UPS were upregulated following RT, HIIT or both; (ii) calpain‐2 protein abundance was upregulated following RT and HIIT, while calpain activity relative to calpain abundance decreased following RT as compared to PRE; and (iii) the observed phenotypic changes pursuant to this training paradigm were recapitulated in vitro using exercise mimetics, corresponding with a decreased rate of MPS in the I+A group. Notably, these results are specific to an untrained population undertaking a specific exercise paradigm consisting of a 7‐week RT block followed by a 7‐week HIIT block, and thus the adaptations observed succeeding HIIT may not apply to those undertaking HIIT in the absence of prior RT. Specifically, it is possible that HIIT elevates certain proteolytic markers as a means of muscle protein turnover rather than strictly catabolism. That is to say, that HIIT without a prior block of RT is not inherently catabolic; however, the combination of upregulated proteolytic markers and a blunted MPS response as compared to RT is not a strong enough stimulus to maintain the muscle size that was attained after a block of RT. It is also important to note that, owing to the nature of these findings as part of a secondary analysis, the results herein should be used as hypothesis‐generating for future research.

Interestingly, 20S proteasome core protein expression and mRNAs *TRIM63*, *FBXO32* and *FOXO3A* were upregulated at POST as compared to both MID and PRE, and 20S proteasome core activity was upregulated similarly at MID and POST. These findings suggest a role for the UPS in the regulation of skeletal muscle while undergoing differential exercise paradigms; however without an in vivo measure of MPS, it is impossible to determine if such upregulation was indicative of enhanced protein turnover (i.e., concomitant increases in MPS) or truly representative of proteolysis. In a study assessing the effects of endurance training on the UPS, Stefanetti et al. (2015) subjected participants to 10 weeks of RT or mixed‐type endurance training, procuring muscle biopsies at acute and chronic time points following training. Notably, the authors found that while untrained, RT and ET increased *FBXO32* mRNA expression, while only HIIT increased *TRIM63* mRNA expression. Furthermore, the authors found that after a single bout of accustomed exercise in the trained state, only ET acutely increased the mRNA expression of *TRIM63* and *FBXO32* from 2.5 to 22 h post‐exercise. Notably, these effects were not observed for muscle MuRF1 protein chronically or acutely. Despite disagreement at the protein level, it is notable that the individuals performing an ET bout presented increases in atrogene expression following a chronic training period. There is additional literature that has assessed adaptations to the UPS following various endurance training protocols in rodents. Cui et al. examined the effects of HIIT versus steady state ET on the expression of MuRF1 and Atrogin‐1 proteins. The authors reported that HIIT induced upregulation of MuRF1 at 4 weeks, and that by week 8 steady state ET produced a more robust increase than HIIT. Additionally, HIIT‐induced Atrogin‐1 protein abundance was lower than steady state ET at both week 4 and week 8 (Cui et al., 2019). Cunha et al. (2012) subjected 5‐month‐old C57BL/6J mice to either 2 or 8 weeks of steady state endurance training and found that *TRIM63* and 26S proteasome core activity was upregulated at 8 weeks. Considering the collective evidence and our findings, it seems plausible that HIIT can induce repetitive upregulations of mRNAs and/or proteins involved in UPS function. It does, however, remain unclear if these increases are a means of enhanced protein turnover without marked proteolysis or if such undulations in UPS involving markers following HIIT contribute to meaningful muscle protein breakdown. It is also important to note that these data represent a single snapshot of muscle status, and therefore transient alterations in the UPS that might influence muscle remodelling were not captured herein. Methodologies involving tracers wherein MPS and muscle protein breakdown (MPB) are measured could lend insight to this unknown.

It is also intriguing that calpain‐2 protein expression was upregulated at both MID and POST, while calpain activity relative to calpain protein abundance was downregulated at MID as compared to PRE. Taken in tandem, we interpret these findings to suggest that while calpain activity was not significantly altered, the potential of calpains to become active in the post‐exercise state is enhanced (e.g., a greater level of calpain‐2 protein abundance might ‘prime’ the calpain system to enhance calpain activity when there is some physiological stressor such as HIIT). This is concordant with studies in both humans and rodents that have examined the acute effects of HIIT (or HIIT mimetics). Place et al. reported that 24 h following an acute bout of HIIT in recreationally active men, maximal force generation was impaired without alterations to motor neuron activation (Place et al., 2015). These authors also reported that ryanodine receptor 1 (RyR1) showed significant fragmentation 24 h following HIIT. Notably, RyR1 is a key protein involved in calcium handling and its fragmentation could lead to calcium‐dependent excitation–contraction dysregulation (Lanner et al., 2010). Fragmentation of RyR1 was concomitant with decreased reactive oxygen species (ROS) metabolic enzymes in humans along with increased ROS in rodents (Place et al., 2015). Importantly, the aforementioned observations occurred in tandem with an increase in calpain activity 30 min and 3 h post‐HIIT perhaps owing to the calcium‐dependent nature of these proteases (Croall & Ersfeld, 2007). Given the findings of these studies, and that the present study was performed in an untrained population, it is possible that transient increases in ROS from HIIT bouts resulted in RyR1 fragmentation and subsequent repetitive episodes of dysregulated calcium handling, thus increasing activation of the calpain system acutely. Indeed, enhanced ROS production has been observed in aerobic and anaerobic exercise (He et al., 2016). Importantly, the time point at which the post‐HIIT biopsy was taken (∼72 h post‐exercise) might have been too delayed to observe calpain activity upregulations in response to increased muscle damage and/or dysregulated calcium handling. Furthermore, it is possible that repetitive upregulations in the calpain system activity played a role in the observed muscle cross‐sectional area decline following a period of HIIT. However, similar to the UPS, it is impossible to be certain of this given that MPS was not measured, and the increases observed herein could be attributed to elevated protein turnover and not necessarily MPB. It is again worth noting that transient alterations in these markers that were not captured might play a larger role in the adaptations observed in this study, and that tracer methodologies to examine MPS and MPB could provide more direct answers to this question.

Our in vitro experiments in C2C12 murine myotubes utilizing ‘exercise mimetics’, in part, recapitulated the phenotypic findings observed in vivo. Notably, 24‐h treatments with IGF1 produced myotube hypertrophy, but these effects were abrogated when the same 24‐h treatment was followed by a 24‐h treatment with AICAR (a known stimulator of AMPK). These findings aligned with what was observed with the IGF1‐treated myotubes demonstrating significantly higher MPS levels than the I+A group. It is additionally notable that statistically significant differences were observed for IGF1‐ versus I+A‐treated myotubes. These in vitro findings suggest that AMPK activation alone can interfere with (and potentially undo) cellular anabolism induced by a prior growth stimulus (i.e., IGF1). While these data suggest a role of an AMPK‐induced abrogation of IGF1‐induced hypertrophy, the roles of these signalling pathways in humans need not be overstated. It is important to note that exercise‐induced increases in phosphorylated AMPK are generally short‐lived (Beaudry et al., 2023), and that changes in muscle IGF1 concentrations are negligible (Nindl et al., 2012; Sterczala et al., 2022). Moreover, these data might be specific to C2C12 myotubes and not necessarily translatable to humans. These data do, however, imply that the loss of skeletal muscle observed in the present study may not have been fully attributable to any of the aforementioned proteolytic pathways. Specifically, the loss of muscle mass observed with 7 weeks of HIIT may have been due primarily to declines in repetitive post‐training MPS rates upon switching from RT to HIIT, as the proteolytic signature associated with HIIT seen in vivo was not seen in these in vitro experiments. In support of this hypothesis, Wilkinson et al. reported that RT increased myofibrillar protein synthesis rates both acutely and chronically, while endurance training did not affect these outcomes (Wilkinson et al., 2008). However, Bell et al. (2015) published contrasting results in older men indicating that a bout of RT and HIIT elevated myofibrillar protein synthesis rates 24 and 48 h post‐exercise. Our in vivo data are limited in that muscle protein synthesis was not determined, and future work using deuterium oxide to delineate integrated muscle protein synthetic rates during weeks‐to‐months of HIIT versus RT would likely provide more insight.

### 4.1 Experimental considerations

Like many studies employing exercise interventions with muscle biopsies, we examined a small number of participants. We were also limited to a fully male sample, and therefore cannot make claims on female adaptations to the given exercise paradigm. Additionally, given the nature of this study as a secondary analysis, our data were limited to only one group. The lack of a control group and/or a comparator group performing dissimilar exercise paradigms limits the overall conclusions drawn. Importantly, a non‐training post RT control would help delineate between HIIT‐ and detraining‐induced proteolysis. As mentioned in the prior section, we are also limited by the ‘snapshot’ like nature of our measures and therefore cannot provide insight into the transient fluctuations that might have occurred in the post‐training state of these participants. Finally, we were unable to collect in vivo muscle protein synthesis measures in this study and therefore cannot determine the role of elevated proteolysis‐related markers in MPB as opposed to muscle protein turnover.

### 4.2 Conclusions

The loss in RT‐induced muscle cross‐sectional area with HIIT may be influenced by increases in muscle proteolytic markers observed in the post‐training period and a reduction in muscle protein synthesis observed in vitro. Future research utilizing tracer methodologies to confirm such contributions is warranted.

## AUTHOR CONTRIBUTIONS

J. Max Michel and Michael D. Roberts conceived the idea for this secondary analysis. Paulo H.C. Mesquita recruited participants, coordinated training sessions and data collections as part of his PhD dissertation. Bradley A. Ruple, Joshua S. Godwin, Paulo H.C. Mesquita and Mason C. McIntosh performed training sessions and data collections. Cleiton A. Libardi, C. Brooks Mobley and Andreas N. Kavazis were critically involved in data interpretation. Daniel L. Plotkin, Joshua S. Godwin, C. Brooks Mobley and J. Max Michel conducted laboratory assays and cell culture experiments. J. Max Michel and Michael D. Roberts primarily drafted the manuscript, all co‐authors provided feedback as well as intellectual contributions. All authors have read and approved the final version of this manuscript and agree to be accountable for all aspects of the work in ensuring that questions related to the accuracy or integrity of any part of the work are appropriately investigated and resolved. All persons designated as authors qualify for authorship, and all those who qualify for authorship are listed.

## CONFLICT OF INTEREST

None of the authors have financial or other conflicts of interest to report regarding these data.

## Data Availability

The data that support the findings of this study are available from the corresponding author upon reasonable request.
